# Clinician perceptions of the impact of ICU family visiting restrictions during the COVID-19 pandemic: an international investigation

**DOI:** 10.1186/s13054-023-04318-8

**Published:** 2023-01-21

**Authors:** Joanne McPeake, Nancy Kentish-Barnes, Emilie Banse, Lynne Anderson, Cecilia Cuzco, Elie Azoulay, Tara Quasim, Pedro Castro, Kathryn Puxty

**Affiliations:** 1 The Healthcare Improvement Studies Institute, University of Cambridge, Cambridge, UK; 2 AP-HP Nord, Saint Louis Hospital, Medical Intensive Care, Famiréa Research Group, Paris, France; 3 Psychological Sciences Research Institute, Université Catholique de Louvain, Louvain-La-Neuve, Belgium; 4 Department of Anaesthetics, Golden Jubilee National Hospital, Glasgow, UK; 5 Medical Lntensive Care Unit, Hospital Clínic of Barcelona, Institut d’Investigacions Biomèdiques August Pi I Sunyer (IDIBAPS), University of Barcelona, Barcelona, Spain; 6 Department of Fundamental Care and Medical-Surgical Nursing, Nursing School of Faculty of Medicine and Health Sciences, University of Barcelona, Barcelona, Spain; 7 Intensive Care Unit, Glasgow Royal Infirmary, Glasgow, UK; 8 School of Medicine, Dentistry and Nursing, University of Glasgow, Glasgow, UK

## Abstract

**Purpose:**

To guarantee the safety of the public, clinicians and patients during the COVID-19 pandemic, hospital visits were severely restricted internationally. There are limited data on the precise impact of these visiting restrictions on Intensive Care Unit clinicians. Our objectives therefore were to explore the impact of family visitation restrictions on clinicians and care delivery and describe innovation alongside areas for potential improvement.

**Methods:**

A qualitative approach using focus groups was employed. We recruited members of the multi-disciplinary team from Spain, France and the UK. Framework analysis was used to synthesize and interpret data.

**Results:**

In total, 28 staff from multiple international sites contributed to data across six focus groups: 12 from the UK, 9 from France and 7 from Spain. In relation to the key aims, we derived four themes: the emergence of new technologies, relationships and rapport establishment, communication challenges and end-of-life care provision. Across each theme, the overarching concepts of clinician emotional exhaustion and emotional distress emerged alongside the negative impact on job satisfaction.

**Conclusion:**

The impact of COVID-19 family visitation restrictions is far reaching. Future research should examine the wider impact of family presence in the ICU.

**Supplementary Information:**

The online version contains supplementary material available at 10.1186/s13054-023-04318-8.

## Introduction

The COVID-19 pandemic has placed unprecedented demands on hospitals and Intensive Care Units (ICUs) internationally [1, 2]. Clinicians have managed complex care situations, alongside their own personal safety, in a highly demanding environment [3]. To guarantee the safety of the public, clinicians and patients, hospital visits were severely restricted [4, 5]. The impact of these practice and policy changes is still emerging.

There has been a proliferation of quantitative literature examining the negative consequences of pandemic care provision on clinicians [6, 7]. The psychological consequences include increased anxiety, signs and symptoms of posttraumatic stress disorder (PTSD) and clinical burnout syndrome (CBS) [8, 9]. For example, one recent European-wide study, undertaken during the pandemic, estimated a prevalence of self-reported burnout of over 50% in ICU clinicians [10]. Specific mechanisms for these psychological sequelae include the impact of managing personal safety, the delivery of palliative care and the challenges associated with family visitation restrictions [11].

Over the last decade, there has been an increased focus on the delivery of family-centred care within ICU [12]. This includes the inclusion of family members in decision making and care provision, facilitated by open and often non-restricted visiting [13]. Yet, there are limited data on the precise impact of these visiting restrictions on clinicians, and what innovations, if any, were successfully adopted to maintain family-centred care during the pandemic.

Therefore, the aims of this study were: explore the impact of COVID-19 family visitation restrictions on ICU clinicians and care delivery and describe innovation alongside areas for potential improvement across three different European countries.

## Methods

This study was approved by the Scientific Officer of the West of Scotland Research Ethics Service as Service Evaluation (UK approval); the Hospital Clínic of Barcelona Medical Research Ethics Committee (HCB/2021/1115) (Spanish approval); and the research ethics board Sud Méditerranée (2020-A00809-30; CNRIPH: 20.03.27.73019) within the framework of the BURDENCOV study (French approval).

All participants provided written informed consent. The Consolidated Reporting of Qualitative Research (COREQ) checklist was used to report findings (Additional file 1: S1) [14].

### Design and setting

A qualitative approach was used to address the study aims. We chose qualitative inquiry as we wished to hear participants describe their experiences in enough detail to enable a comprehensive understanding of the interplay between family visitation policies and clinician experience. Focus groups were chosen as a method of data collection as the interaction between those involved is thought to help participants consider and reflect upon aspects of daily life (and work) that are sometimes taken for granted [15].

Clinicians were recruited from three international sites to understand if staff experiences differed across international contexts. Details of the international policy related to hospital visitation are provided in Table 1. Interviews were undertaken in the local language and translated into English. Participants were recruited through advertising this research in staff areas and via personal solicitations; staff interested in participating were then given the option to attend a focus group. We aimed to recruit a diversity of clinicians to fully understand the impact of family visiting restrictions across the entire MDT.

**Table 1:** Visiting policies across the international contexts

Country	Date	Guidance
UK	Pre-COVID-19	Open visiting for all ICU patients
	March 2020	UK Lockdown. One visitor at the end of life, visitor then follows COVID exposure guidelines and self isolates following the visit
	July 2020	One named designated visitor plus additional visitor at end of life
	August 2020	Two designated visitors plus additional visitor at end of life
	September 2020	End-of-life visiting only
	April 2021	One named visitor
	July 2021	Two visitors
	August 2021	Full person-centred visiting
	January 2022	End-of-life visiting only
	March 2022	One interchangeable visitor per day
	May 2022	Full patient-centred visiting
Spain COVID ICU	Pre-COVID-19	3 times a day (7:15–7:45; 13:00–14:00; 20:00–21:00), any visitor, maximum 2 at the same time. Exceptions were made depending on the patient
	March 2020	No visitors allowed. Exceptions were made: one visitor at the end of life. Information was provided by phone
	June 2020	No visitors allowed. Exception included patients admitted for more than 8 weeks
	November 2021	One visitor 1 h (13 h or 20 h). Exceptions were made depending on the patient
	May 2022	2 times a day (13:00–14:00; 20:00–21:00), one visitor. Exceptions were made depending on the patient
Spain Non-COVID ICU	Pre-COVID-19	3 times a day (7:15–7:45; 13:00–14:00; 20:00–21:00), any visitor, maximum 2 at the same time. Exceptions were made depending on the patient
	March 2020	Spain Lockdown. One visitor at the end of life
	April 2020	One visitor 1 h (13 h or 20 h)
	May 2021	2 times a day (13:00–14:00; 20:00–21:00), any visitor, maximum 2 at the same time in the room
	July 2021	One visitor 1 h (13 h or 20 h)
	September 2021	2 times a day (13:00–14:00; 20:00–21:00), one visitor
	October 2021	3 times a day (7:15–7:45; 13:00–14:00; 20:00–21:00), any visitor, maximum 2 at the same time. Exceptions were made depending on the patient
	November 2021	2 times a day (13:00–14:00; 20:00–21:00), one visitor
	December 2021	One visitor 1 h (13 h or 20 h)
	February 2022	2 times a day (13:00–14:00; 20:00–21:00), one visitor
	May 2022	3 times a day (7:15–7:45; 13:00–14:00; 20:00–21:00), any visitor, maximum 2 at the same time. Exceptions were made depending on the patient
	June 2022	2 times a day (13:00–14:00; 20:00–21:00), one visitor
	September 2022	3 times a day (7:15–7:45; 13:00–14:00; 20:00–21:00), any visitor, maximum 2 at the same time. Exceptions were made depending on the patient
France	Pre-COVID-19	Open visiting for all ICU patients
	March 2020	France Lockdown: No visitors allowed in the hospital. Saint Louis ICU allows one visitor at the end of life. No waiting room
	April 2020	The ICU at St Louis decides to allow one visitor/patient/day. In end-of-life situations, more than one visitor allowed as long as the visitors do not come at the same time. No waiting room
	June 2020	Open visiting for all ICU patients during the day. However only one visitor at a time. No waiting room
	October 2020	Open visiting for all ICU patients as before COVID

Focus groups were facilitated by experienced researchers and were undertaken in-person (in the hospital setting) and virtually. Researchers involved in focus group facilitation had extensive experience of qualitative data collection. Within this analysis, data saturation was defined as the point at which linking the concepts of two consecutive focus groups revealed no additional insights [16]. This was determined by the research team via iterative discussion across international sites.

A semi-structured topic guide was developed a priori (Additional file 2: S2); questions were open ended, and participants were encouraged to explore issues they considered relevant. Questions were reviewed by participants for clarity before utilization. Demographic data were collected from participants which included gender, age, profession and number of years of experience in critical care.

### Data analysis

Data were analysed using thematic analysis, informed by framework analysis [17]. This analytical framework allows structured and systematic analysis of data and has been used in previous critical care research [18, 19]. The data were systematically analysed against the research aims: explore the impact of family visitation restrictions on care delivery and clinicians; describe innovation and explore areas for potential improvement. The framework analysis technique was used to analyse data across these concepts, through seven stages: (1) Transcription; (2) Familiarization; (3) Coding; (4) Developing a working analytical framework; (5) Applying the analytical framework; (6) Charting data into the framework matrix; and (7) Interpreting the data [17].

Two researchers (JM and KP) independently undertook preliminary sweeps of the data, and initial codes were identified. The codes were checked against the study aims, resulting in the development of a preliminary coding framework. These initial codes were used to develop a thematic framework by grouping themes, which linked concepts. Thematic sets were then created and organized into a framework matrix to create the final themes (Additional file 3: S3). Following the creation of this matrix, researchers examined potential international differences. Key quotes to support findings were extracted by JM and KP.

Member checking with participants was undertaken. Member checking, also known as participant validation, is a technique for exploring the credibility of results. Data or results are returned to some participants to check for accuracy and resonance with their experiences [20]. Participants who had agreed to ongoing contact with the research team were involved in this process (n = 3). Participants involved in the member checking process were randomly selected for involvement in this step of the data validation process.

## Results

In total, 28 staff from three international sites contributed to data across six focus groups. These participants represented multiple ICU professions including nursing, medicine, physiotherapy, pharmacy and health care support workers. Focus groups were undertaken between June 2021 and July 2022. Each focus group lasted between 45 and 80 min. Participant demographics are detailed in Table 2. After iterative discussion across the research team, no new themes emerged following these focus groups and data saturation was achieved.

**Table 2:** Demographics of focus group participants

Demographic	n (%)
Participant Region:	
UK	12 (43)
France	9 (32)
Spain	7 (25)
Gender	
Female	21 (75)
Male	7 (25)
Age, Years, Median (IQR)	39 (IQR:32–47)
Professional background	
Nurse	10 (36)
Medicine	10 (36)
Physiotherapy	3 (11)
Healthcare Support worker	2 (7)
Advance Practitioners in Critical Care	2 (7)
Pharmacy	1 (3)
Years of ICU experience	
Less than 5	6 (21.5)
5–10 years	6 (21.5)
11–15 years	8 (29)
16–20 years	4 (14)
20 years +	4 (14)

In relation to the aims, we derived four themes from the data: the emergence of new technologies; relationships and rapport establishment, communication challenges and end-of-life care provision (Table 3). Within each theme the impact on clinicians, care delivery, innovation and potential improvements were delineated. Across each theme, the overarching concepts of emotional exhaustion, emotional distress and low job satisfaction emerged. Following a robust examination, we found no differences across international sites, indeed the findings mapped in an identical manner internationally. A selection of representative quotes across international sites is presented in Table 3.

**Table 3:** Representative quotes illustrating key themes

	Staff	Care delivery	Improvements and innovations
Emergence of new technologies	The phone calls were one of the most stressful things…Trying to reassure them, without actually knowing anything… There was not any reassurance I felt I had to give… I think there was a lot of anxiety just around phone calls and communication We were also able to find alternatives, that is to say give online meetings and that was really a way for us to be able to at least give something	It put a huge barrier on communication At worst it was anxiety provoking for families to see the person. A first visit to ITU seeing all that is destabilising, so on the screen … oh dear… with interference because the networks are rubbish. You didn’t hear well …. It was stressful	The majority of people didn’t have passwords and trying to get people to explain it to you, to show it to you. They are busy The initiative of tablets appeared, to make video calls with families, which as an initiative can be super-good and was very necessary, but at the level of workload, it was often incompatible… there were no hands or people to dedicate the time to it
Relationships and Rapport Establishment	The family brought the patient to life for us It meant much more wear and tear to care for patients, no longer at the level of techniques or things, but at the ‘human’ level No family, no relatives and everything so it was often the nursing teams who had to make up for this deficit when we went to zero visits	We went from being totally holistic and patient-centred to just very task oriented FP: There was also a feeling of disconnect from reality for the families	The volunteer service… was really helpful, where people were able to drop the things off, and then the volunteers were bringing them up. That felt like a really big step, even being able to have a few personal items from home.” Flexibility of schedules was something that could be allowed more
Communication Challenges	It is not the same to talk on the phone than to talk in the face-to-face situation... I think especially the proximity, the flexibility and the face-to-face situation.. it always makes it more human It helps us to know what the patient was like before … We learn things… Medications, treatments, their way of life ….. how independent they are … All that	You had to really make an over effort to explain things in great detail so that they could get an idea, and explain the steps, and without creating like too much hope… It is difficult to make the family understand what is happening without them being present.” I felt there was a total lack of understanding on the phone, and that I couldn’t help the families at all, on the phone	Someone on the team has to dedicate themselves to family. Knowing that they can’t get in or out in any way, that they can’t take off their robes in any way Doing some, almost simulation training, of communication over video
End-of-Life Care	I actually found the end-of-life stuff the hardest…I think, there are just so many different situations, where we weren’t allowed to let families in… I think the emotional burden of that was massive But it was a shock from 24 h a day to 0 and after, except for the people who were dying …. No, not at all, no ……they died alone …yes, they were alone	Only having visitors at the end of life made me think a bit differently about decisions to change the focus of care to palliation. I felt I had to make sure I got it 100% right. There was only going to be one chance, they definitely had to be dying. They were only going to be allowed to visit once. If they didn’t die in that visit then they weren’t allowed back in because they were isolating.” I sometimes had to arrange video and phone calls at the same time, they all wanted to say goodbye. I would bring the phone to the patient’s ear. It was not easy. Patients would not be able to speak, so we have to translate, in our own words. The most difficult is finding the right words	I once have a doctor whispering to me: ‘I told them they could all come in, the 4 of them’. I just said minutes before, that they could only come 2 by 2, we couldn’t have more at the same time, given the organisation of the ward. As a consequence, we are not credible. Yes, that is another consequence of having mixed, blurred messages: the lack of credibility The restrictions, they were much longer in the other departments and much more draconian

### Emergence of new technologies

The introduction of new technologies for the facilitation of family communication was discussed. The rapid pace at which technology was implemented caused anxiety for participants. Anxiety was due to a perceived lack of governance arrangements, alongside worries about how families may feel when viewing their loved one during video calls.

‘…*but if the patient had been in the prone position, I tried to find an angle that wouldn’t shock the family… and taking those pictures, it stayed with you.’*

Participants also described anxiety around access to technology from the family perspective. Inequalities in access to technology, especially with elderly patients, caused apprehension with staff.

‘*I really felt for the generations in the family. They were saying ‘I don’t know if I have got that type of phone, or I’ll need to wait until my granddaughter comes round.’ I was anxious about that. ’*

Across the focus groups participants described how the ‘*non-technical’* skill of communicating with families became a ‘*technical* *skill’*. Organic conversations within ICU, which staff described as being important to delivering consistent care, were formalized into ‘factual’, prescribed telephone conversations:

‘*I think the most horrible part from my perspective is that it became a very cold situation… if a patient dies, it is very painful and ugly, but when you see the family is when it hurts on a personal level. This by phone, because it is done as something more mechanical and cold. It was horrible, it was cold.’*

Potential improvements in relation to virtual visiting were explored. Improvement activities were primarily focused on education and training needs of staff, alongside the provision of logistical requirements such as password access. Participants highlighted that training in undertaking virtual visiting required as much focus as *‘technical’ caring duties*:

*‘There was nothing about how we were going to communicate with families. We were practicing proning, we were practicing all the kind of treatments and the things we were going to do, but there was no discussion about how we were going to communicate with families.’*

### Relationships and rapport establishment

Participants highlighted the crucial role which families play during ICU admission. For example, a clinician highlighted the importance of family relationships in care delivery:

‘*It is like cleaning a patient, maybe it is a burden, but in the end, it is for the wellbeing of the patient. The family is the human part that is not a burden. I think it is something necessary. Does it take time? Yes. Do we have to adapt to that? Yes. Are they sometimes difficult? Also, a lot. But it is necessary. Super-necessary.’*

During the initial stage of the COVID-19 pandemic, participants described a move from personalized family interactions to ‘*transactional*’ relationships. This loss of relationship and rapport had several negative consequences for clinicians including increased anxiety about the care family members were receiving and the creation of a ‘*cold*’ working environment:

‘*It suddenly became very cold…it was very strange frankly, it was a bit dehumanizing…. it was a bit glacial.’*

This change in relationship dynamic between staff and family members was also perceived to have a negative impact on care delivery. Participants described how care became depersonalized, with a loss of person-centred care:

‘*Even if someone had been sedated for a few weeks, I feel like you were able to get a really good picture of their homelife. Their families would bring up photographs and tell you stories about who they were. If that person woke up, you felt that you had a relationship with them because of their family. With COVID, it felt like everybody was just the same…You didn’t know who they were as people, it made it really difficult to be as empathetic and provide that side of care.’*

The creation of tools to improve relationships and rapport between patients, families and staff were discussed. In addition to the introduction of virtual visitation, these included the use of electronic ‘*what matters to you tools’* as well as individualised solutions such as personalized family messages and recordings:

‘*A family put up videos, recorded videos of themselves talking to the patient, just saying reassuring things, hearing familiar voices and playing music they were familiar with. They uploaded the video and you could have it playing at the bedspace continually… I remember thinking it was really good.’*

### Communication challenges

Across international settings, participants described the challenges which visiting restrictions brought to communication. One of the most challenging aspects was the difficulty in describing the clinical ‘*situation*’ without family members present. Participants recounted that pre-pandemic, family members would often be at the bedside much of the day and could comprehend via formal and informal conversations, as well as through direct visualization, the progress, or lack of progress which a patient was making. As such, communication without families present was more difficult:

‘*They (families) need to be informed and very often need to be reassured in a visible and concrete way…I wait for the family to be there to do a session so that the relative can see the evolution and the progress.’*

The challenges with communication were perceived to have a direct impact of care delivery. Not having families present to provide commentary and support, made care delivery challenging. A physiotherapist described the negative impact of fragmented communication:

‘*When patients were fit enough to begin rehab, they tended to be at a much lower physical ability level. The global effect on the patient was huge….you had the psychological distress, there were huge chunks of their life missing. They didn’t have their family with them, they had no idea what was going on…Getting patients to engage without that sense of familiarity was an added challenge on top of their physical limitations.’*

The contribution of family members in supporting communication and care during rehabilitation was also discussed within the context of delirium management:

‘*…most of them are super disorientated, agitated and very restless and I think that in those circumstances seeing a familiar face, a relative, always helps. Apart from the fact we were strangers…with the mask…I mean we were in disguise.’*

Innovations and improvements to support communication were described. One area of innovation included the development of local *‘environment’* films. These included be pre-recorded films describing the ICU environment, alongside noises and equipment, which families could view, to allow contextualization and visualization of how care was being delivered. This was utilized in place of in-person visits to ICU where families would encounter the machines and technologies present in an ICU setting and thus facilitate improved communication during telephone conversations with staff.

*‘I think it would have been helpful if there was something we could have sent them electronically…this is the ventilator, this is the computer, this is the monitor, these are the infusion pumps…this is what to expect in ICU.’*

### End-of-life care

The provision of end-of-life care was explored in-depth. In relation to the impact for staff, there was the need for a change in roles for clinicians. Participants across all disciplines acknowledged that this was particularly difficult for nursing staff; they described nurses had to adopt the role of family members in palliative situations, as relatives were unable to be present in the hospital.

‘*Sometimes we would feel a certain transference. We would say ‘if it was someone in our family, wouldn’t we like that someone in the ward stayed with him or her as she passes away? Can’t we make that gesture at least?*’

Changes to the delivery of care *‘rituals’* during palliation were also described by participants. A participant from France spoke of these changes:

*‘For palliative cases, we would open the doors to families, put a bed in, try to support them… and it is exactly the opposite, people are dying and we shut the doors, it is a bit bewildering, surprising to do the opposite.*’

A lack of standardization across visitation restrictions and inconsistent implementation locally caused anxiety and frustration. In relation to improvement activities, staff described the importance of a consistent approach as being key to improving and delivering care:

‘*That’s when our ethical dilemma begins: ‘okay I will let you in, but because it me, I assume responsibility of that, but tomorrow maybe not.’*

Interestingly, a small number of staff also discussed how family visiting restrictions may have been beneficial to the provision of end-of-life care and care provision more widely during the pandemic. This feeling appeared to be rooted in the volume of death which was experienced within the ICU environment during the pandemic, alongside the nature of care, such as frequent proning. For example, one participant discussed how families may have been *‘traumatised’* by the ICU environment during this time:

*‘I almost found it easier not to have relatives because there were people dying so often and they just didn’t look like themselves. Did we actually save them from something in a way? How hard would it have been to come up daily… to have seen their loved one proned all the time, and when we would unprone they became ill so quickly, the rush that happens after all of the staff appear, actually, would that have traumatised them?’*

### Staff well-being

**Fig. 1:**
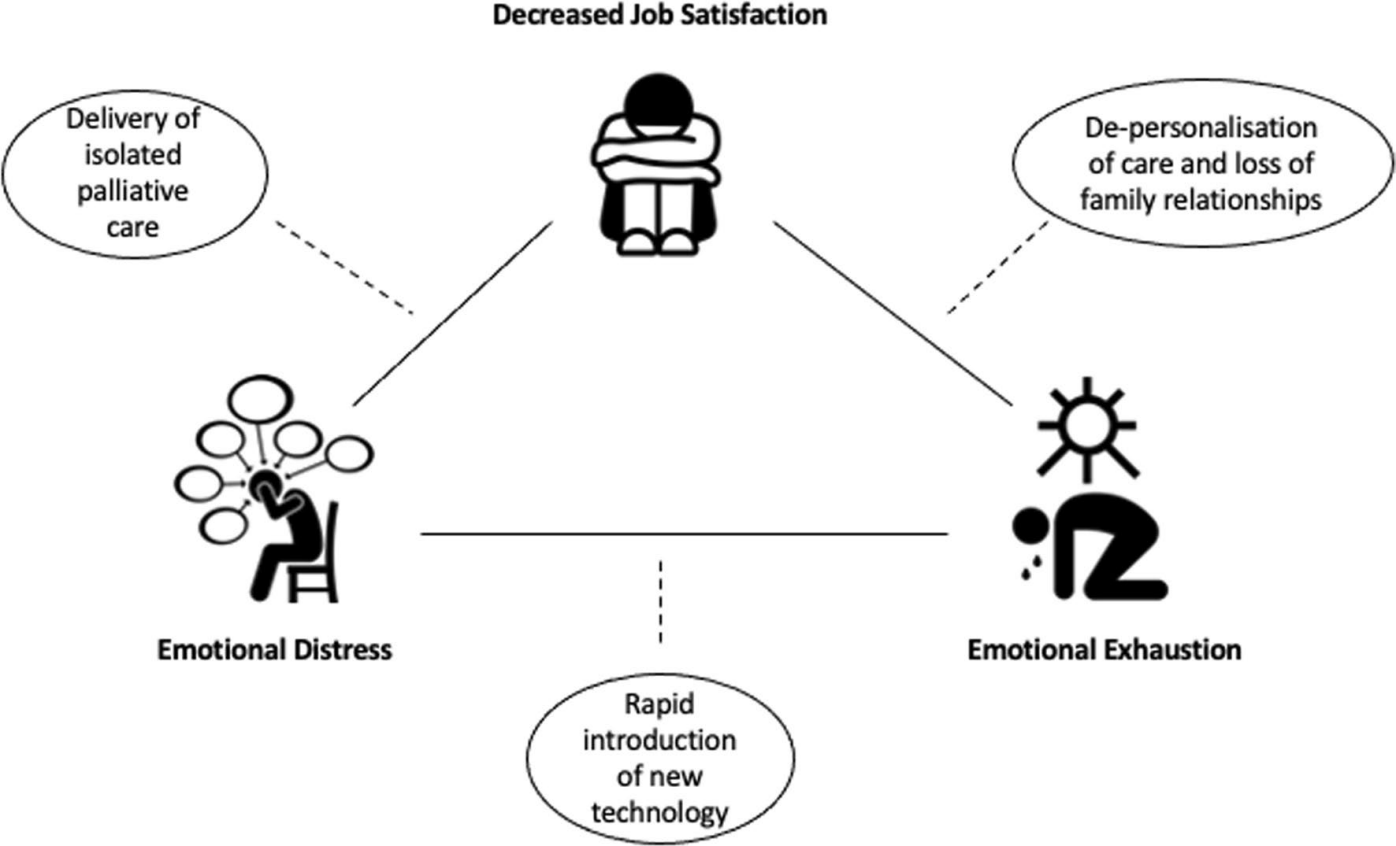
Conceptual Figure describing the impact on staff well-being of restricted visiting arrangements

Across theme generated, there was the manifestation of emotional exhaustion, emotional distress, and loss of job satisfaction for clinicians (Fig. 1).

Emotional exhaustion manifested in several ways; most notably staff described feelings of fatigue and being overwhelmed. A participant described the impact of the introduction of new communication strategies, to ensure that families had access to their loved one and the impact that this had on clinicians:

‘*….not only giving them a clinical update, but then trying to get hold of their email, trying to sort out technology. I just found it a bit overwhelming on top of the job we already do.’*

The negative, longer-lasting emotional consequences that pandemic care provision and visiting restrictions had on staff well-being, alongside subsequent emotional distress, also emerged:

*‘For me the satisfaction is that we get people better, although there are people who do not improve and suffer. But for me what has happened with the pandemic, the management of all that pain, I think many of us carry it. I have not been able to manage it.’*

In relation to end-of-life care, staff also described feelings of guilt and frustration, which led to distress. Clinicians delivered heartbreaking news to families, in parallel with enforcing visiting restrictions:

‘*We were saying ‘yeah you can come up, but you are going to get exposed to COVID, which your loved one is going to die from, and you are going to have to isolate afterwards and not be able to get support from anybody.’*

Visitation restrictions and the consequences of these restrictions appeared to result in a loss of job satisfaction. One participant described how their role felt without the presence of families:

*‘It was a feeling similar to working on a production line in a factory, without the contact of families…it was like a factory job.’*

## Discussion

This qualitative analysis described the impact of pandemic visitation restrictions had across a diverse range of ICU clinicians, from multiple European settings. It has demonstrated those visitation restrictions implemented influenced care delivery in the ICU. The development of innovation was described, including the use of virtual visiting technology, as well as areas for future quality improvement efforts. Across international sites, clinicians described feelings of emotional distress and exhaustion due to the implementation of restrictions. The absence of visitors also appeared to have a negative impact on job satisfaction.

The importance of family input into the provision of ICU care is not new [21, 22]. Prior to the pandemic, research focused on the type of visitation arrangements (*flexible vs fixed*) and demonstrated the impact on rates of delirium and ICU acquired infections [23]. The present findings extend this previous knowledge by delineating the impact that family visitation has on the entire ICU system, including care delivery and clinician well-being. Indeed, the findings of this research would suggest that family members may be crucial members of the ICU team.

While the findings of this study do not provide unequivocal evidence, they should be used as a foundation for further empirical research, exploring the full and diverse contribution of families in the ICU environment.

Recent evidence has shown that family members appreciate and benefit from virtual visiting and the technology which has been implemented to facilitate virtual visiting [24]. However, similar to previous research this analysis found discordant views about the use of technology from the clinician perspective [25]. Participants described anxieties around the introduction of this technology including governance arrangements and the ability to undertake filming due to logistical requirements. Interestingly, this analysis extends these insights by highlighting that some family members appeared to struggle with access due to digital literacy and access to appropriate technology. There have been several reports describing the negative impact which virtual clinic attendance has on health inequalities, with those from more socioeconomically deprived areas, and older adults most likely to be disadvantaged [26, 27]. To our knowledge, this evidence is not available in relation to virtual visiting for family members. During subsequent development, it is crucial that health inequalities are not exacerbated through this innovation. Future research should examine this technology with specific emphasis on evaluating the intervention across the socio-economic spectrum.

Despite differences in international visiting restriction policies, we found no differences across the sites included, with the mechanisms for the negative impact on clinician well-being being mapped in an analogous manner. The researchers did not ask participants to discuss particular timeframes in relation visiting restrictions; broad experiential questions around restrictions were asked. Other factors may have therefore contributed to our findings, which were not captured via the focus group approach, for example, pandemic care hospital policies. However, it might also be the case that any type of restrictions may have consequences for care delivery. Future national and local policy should be mindful of the multiple effects which could arise from visiting restrictions.

Participants spoke in detail of the negative consequences which the restrictions had on their ability to provide high-quality, person-centred care. This contributed to depersonalization of care and subsequent emotional distress for clinicians. Emotional distress, fatigue and poor job satisfaction have been identified as key contributors to CBS [28, 29]. Previous literature has objectively measured a high rate of CBS in ICU clinicians during the pandemic, with visitation policies cited as a key driver [7, 30]. This research highlights the crucial need for clinician psychosocial support, to address the potential outcomes of CBS. CBS is not benign and has multiple clinical implications including decreased patient satisfaction and care quality, alongside increased rates of staff turnover, with obvious implications for patient safety [29]. When addressing CBS therefore, it is crucial that managers and policy makers focus efforts of developing a resilient system which provides environmental and cultural protection from the causes of CBS, and not focus primarily on individual staff psychology and coping skills [31]. Uniquely, this present research develops tangible mechanisms by which CBS could be mitigated including adequate training in relation to technologies and the provision and implementation of consistent visitation policies. More novel techniques such as reconnection with patients and family members following hospital discharge have also been cited as potential mediators of CBS, with bidirectional benefits for patients [18, 28, 32]. Reconnection could be facilitated via patient follow-up, informal visits back to ICU, or staff and family joint events. Future research should examine these low-cost strategies in the context of CBS.

Interestingly, participants did not focus on safety issues such as infection control, which were the primary purpose of restrictions. Moreover, although a small number of staff did describe some potential positive consequences of restrictions, due to the ‘*traumatic’* nature of the care environment, the primary focus across the three international contexts was around the emotional and relational damage which emerged because of restrictions. This finding might be due, in part, to the timing of the data collection, especially with the establishment of vaccination programmes and stringent public health measures, alongside the subsequent decline in ICU mortality [33]. It might also be that that the perceived negative consequences which emerged due to visiting restrictions were viewed as a greater risk to family and patient well-being, than issues such as infection transmission. Policy makers should carefully consider the multiple effects of restrictions and implement tailored, balanced mitigation strategies in response to these if this situation arises in the future.

Strengths of our study include the inclusion of rich perspectives from a broad range of professionals, across international sites. There are limitations to these data. Although the use of focus group data collection has multiple advantages, this approach may be subject to bias, as participants are motivated to take part. Those who facilitated the interviews also contributed to the analysis and presentation of data. The researchers took multiple steps to ensure that the validity of the findings were not compromised in relation to this, such as the use of member checking; however, other interpretations of the data might have been found. Finally, we did not explicitly capture or measure CBS or emotional distress. As such, this interpretation warrants further investigation.

## Conclusion

In conclusion, the impact of family visitation restrictions is far reaching. We identified that the restrictions had an impact on staff well-being and job satisfaction alongside a negative impact on care delivery. Potential innovations to develop care delivery and staff well-being were identified and included adequate training in relation to technologies and the provision and implementation of consistent visitation policies.

## Supplementary Information

**Additional file 1. S1:** COREQ checklist.

**Additional file 2. S2:** Focus group interview schedules.

**Additional file 3. S3:** Qualitative analysis coding development

## Data Availability

A de-identified dataset and the study protocol may be made available to researchers with a methodologically sound proposal, to achieve the aims described in the approved proposal. Data will be available upon request following article publication. Requests for data should be directed at joanne.mcpeake@glasgow.ac.uk to gain access.
