# Supplementary material for: Clinician perceptions of the impact of ICU family visiting restrictions during the COVID-19 pandemic: an international investigation
Source: Crit Care. 2023 Jan 21;27:33. doi: 10.1186/s13054-023-04318-8 (PMC9862209; doi:10.1186/s13054-023-04318-8)
Supplement: Supplementary file 2 — Additional file 2. S2: Focus group interview schedules. [file 13054_2023_4318_MOESM2_ESM.docx]

**Focus Group Schedules/Guides**

**Version 1 25^th^ of February 2021**

**Staff (Focus Group)**

1. Can you tell me about ‘usual’ interactions with relatives before the COVID-19 visiting restrictions?
2. Could you describe how you in your professional role interacts with visitors in the ICU?
3. How do you feel families were involved in the care of patients in the ICU prior to COVID-19?
4. Can you tell me about visitation policies before the COVID-19 pandemic and how they changed?
5. Can you tell me how you feel how patients are affected by the lack of visitors during the COVID-19 pandemic?
   1. What were the drawbacks of this situation?
   2. Did you feel there was positives to come from the lack of visitors during the pandemic?
   3. Did you feel there was any positives for the patients?
   4. How did you think relatives found this?
   5. How did you feel about the visitation policies in relation to your workload (including track and trace as the policies changed)?
6. How did communicate with relatives?
   1. How did you find this experience?
   2. Is this normal for you prior to COVID-19?
7. How well do you know your patient?
   1. Do visitors change how you know your patient? Can you tell how?
   2. Do you feel that not having visitors has affected how well you know your patients? Can you tell me a little more about this?
8. Thinking about your recent experience, how has COVID-19 has changed how you feel about visitors in the ICU?
9. Is there anything about your experience of working in the ICU during COVID-19 in relation to visitors and visiting that you think I should understand better?
10. Is there anything you would like to ask me?
11. Would you like to be informed of the results of this study?
