# Supplementary material for: Clinician perceptions of the impact of ICU family visiting restrictions during the COVID-19 pandemic: an international investigation
Source: Crit Care. 2023 Jan 21;27:33. doi: 10.1186/s13054-023-04318-8 (PMC9862209; doi:10.1186/s13054-023-04318-8)
Supplement: Supplementary file 3 — Additional file 3. S3: Qualitative analysis coding development [file 13054_2023_4318_MOESM3_ESM.docx]

**S3: Qualitative Analysis Coding Development**

*Stage 3: Coding*

**The following section highlights the initial coding developed independently, then in collaboration between JMcP and KP, following initial sweeps of the focus group data.**

Emotional Burden for staff

Emotional Fatigue

Increased care requirements, including changes to roles (family)

Trauma/palliation

Guilt/fear

Decreased work satisfaction

Long-term impact

Inability to care for relatives

Specific skill set development

Technical/non-technical skill development

Technology personalisation

Technology anxiety/use/staff/training/time/? Added role

Prioritisation of family care

Innovation in technology development including distinct advantages

Policies around communication/application/rule breakers/ consequences/ distress

Volunteer service roles

Inequalities ? exacerbated by technology application

End of life care/technology/trauma/relationship development/trust

Transactional relationships with families

Loss of feedback and relationships- impact on staff wellbeing

Anxiety- who is caring for families

Anonymous patients/ person centred care delivery/ depersonalisation

Loss of MDT approach and skills delivery- especially in relation to palliation

Loss of ‘warmth’ palliation/care delivery/environment

No visualisation of care. The journey difficult to comprehend

MDT input (pharmacy PMH, physio motivation)

Impact on patient psychological wellbeing

Loss of ability to deliver good psychological care

Patient social isolation/loneliness

Delirium management- impact of family presence/impact/negative consequences

Rehabilitation without personalisation

Consistency in care without families- challenges

Global impact on patient care

End of life rituals.

*Stage 4: Developing an analytical framework*

**Following the initial raw coding stage, the transcripts were reviewed once more independently.**

**Following this, the codes were checked against the study aims (care delivery, clinician impact and innovations and improvements).**

**Following this step, a preliminary coding framework was developed which included:**

- **Relationships and Rapport establishment**
- **Emergence of new technologies**
- **Communication challenges**
- **End of life care**

**Across each themes developed, the overarching concepts of emotional exhaustion, emotional distress and job satisfaction emerged.**

*Stages 5-6: Developing and Applying the analytical framework*

**In collaboration across the authorship team, the following analytical framework was developed and applied by JM and KP. Thematic ‘sets’ were then developed and organised into our framework matrix (Table 1). Of note, some codes could be placed alongside multiple themes. For the purpose of clarity, we have placed them next to a single theme at this stage of the framework analysis. This may have been altered slightly during the interpretation and presentation of our results.**

**A robust examination was undertaken to examine any international differences (verified by EB) at this stage.**

*Stage 7: Interpretation*

**The presentation of representative quotes are shown in the main paper. This presentation, alongside the discussion section of the main paper, represents the interpretation stage of our qualitative analysis.**

| **Issue** | **Theme** |
| --- | --- |
| ***Care Delivery*** | |
| Reduced ability to support families | Relationships |
| Inability to confirm PMH or drug history | Communication |
| Inability of the family to “bring the patient to life” | Relationships |
| Family not part of the ICU journey so didn’t see the clear patient trajectory or able to fill in the blanks for the patient following ICU | Relationships |
| Depersonalisation of patient | Relationships |
| Communication barrier of technology | Technology |
| Lack of nonverbal cues of understanding | Communication |
| Family inability to access virtual communication tools reduced ability for us to communicate | Technology |
| Reduced MDT family discussions as only one person on the phone | Communication |
| EOL visiting decisions/ timings | EOL |
| Family regrets at not visiting at EOL | EOL |
| Fewer family updates | Communication |
| Family updates from clinicians not directly involved in the patient care | Communication |
| Less rapport, more factual transactional interactions with family | Communication |
| Too much burden on NOK | Relationships |
| EOL experience via phone or video | Technology |
| Relatives not given ICU information leaflet | Communication |
| Management of delirium difficult | Relationships |
| Lack of relationship with patient due to limited family interaction | Relationships |
| No combined physiotherapy with families | Relationships |
| Reduced spiritual needs addressed | Relationships |
| Family unable to frame the patient experiences | Relationships |
| Lack of security for patients from family absence | Relationships |
|  |  |
| ***Impact on clinicians*** | |
| Anxiety over self awareness on screen | Technology |
| Lack of familiarity with technology | Technology |
| EOL “horrendous” and “soul destroying” | EOL |
| Emotional instability | Relationships/ Burnout |
| Lack of MDT approach to EOL discussions distressing | Communication |
| Trauma from feelings of providing poor family care, felt I was letting them down | Relationships |
| Confusion regarding “rules” of visiting | Communication |
| Concern about possibility of complaints due to poor communication | Communication |
| Stress over getting the tone right on phonecalls | Communication |
| Lack of passwords or training to use technology overwhelming | Technology |
| Active involvement in death | EOL |
| Taking on the role of the family | Relationships |
| PTSD from virtual deaths | EOL |
| Concern of patient confidentiality and use of videos | Technology |
| Dehumanising of patient care | Relationships |
| Heroes and angels | Relationships |
| Guilt over lack of family access at EOL | EOL |
| Patients dying alone without family | EOL |
| Frustration over inflexible visiting policy | Relationships |
| Doing the best we could | Relationships |
|  |  |
| ***Innovation and Improvements*** | |
| Ensure passwords for all | Technology |
| Adequate training on new technologies | Technology |
| One way video calls of patient for families to see | Communication |
| One way videos of families for patients to watch | Communication |
| Interactive videos for clinicians/ patients to interact with families | Communication |
| Staff buddy system | Relationships |
| Consistent staff teams | Relationships |
| Patient and family follow up appointments | Relationships |
| Bereaved family follow up appointments | Relationships |
| Communication training | Communication |
| Formal visiting group to agree on “rules” | Communication |
| Clarity over the role of technology | Technology |
| Adequate amount of equipment | Technology |
| Posting the ICU information leaflet | Communication |
| Family posting or delivering patient photos or items | Relationships |
|  |  |

**Table 1: Thematic sets used to create the framework analysis presented in this paper**
